# Genetic contributions to bipolar disorder: current status and future directions

**DOI:** 10.1017/S0033291721001252

**Published:** 2021-10

**Authors:** Kevin S. O'Connell, Brandon J. Coombes

**Affiliations:** 1 Division of Mental Health and Addiction, NORMENT Centre, Institute of Clinical Medicine, University of Oslo, Oslo University Hospital, 0407Oslo, Norway; 2 Department of Health Sciences Research, Mayo Clinic, Rochester, Minnesota, USA

**Keywords:** Mood disorder, affective disorder, psychiatric genetics

## Abstract

Bipolar disorder (BD) is a highly heritable mental disorder and is estimated to affect about 50 million people worldwide. Our understanding of the genetic etiology of BD has greatly increased in recent years with advances in technology and methodology as well as the adoption of international consortiums and large population-based biobanks. It is clear that BD is also highly heterogeneous and polygenic and shows substantial genetic overlap with other psychiatric disorders. Genetic studies of BD suggest that the number of associated loci is expected to substantially increase in larger future studies and with it, improved genetic prediction of the disorder. Still, a number of challenges remain to fully characterize the genetic architecture of BD. First among these is the need to incorporate ancestrally-diverse samples to move research away from a Eurocentric bias that has the potential to exacerbate health disparities already seen in BD. Furthermore, incorporation of population biobanks, registry data, and electronic health records will be required to increase the sample size necessary for continued genetic discovery, while increased deep phenotyping is necessary to elucidate subtypes within BD. Lastly, the role of rare variation in BD remains to be determined. Meeting these challenges will enable improved identification of causal variants for the disorder and also allow for equitable future clinical applications of both genetic risk prediction and therapeutic interventions.

## Definition of illness

**Fig. 1.**
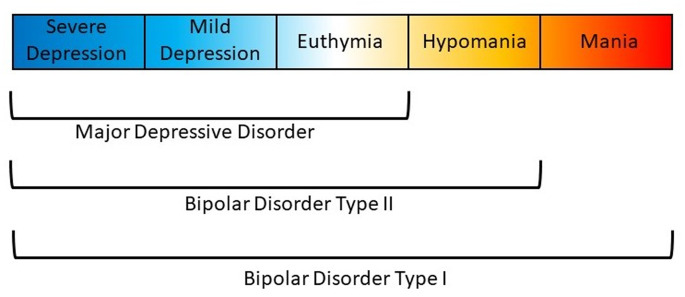
Polarity of symptoms for bipolar disorder subtypes. Bipolar disorder type I is characterized by at least one manic episode. Bipolar disorder type II is characterized by at least one depressive and one hypomanic episode during the lifetime, but no manic episodes. Major depressive disorder does not include episodes of hypomania or mania.

Affective disorders are classified along a spectrum from unipolar depression to bipolar disorder (BD) type II and type I (Carvalho, Firth, & Vieta, 2020; Grande, Berk, Birmaher, & Vieta, 2016). The presence of recurring manic or hypomanic episodes alternating with euthymia or depressive episodes distinguishes BD from other affective disorders (American Psychiatric Association, 2013; World Health Organization et al., 1992). BD type I (BDI) is characterized by alternating manic and depressive episodes (Fig. 1). Psychotic symptoms also occur in a majority of these patients which may lead to compromised functioning and hospitalization. The Diagnostic and Statistical Manual of Mental Disorder, Fifth Edition (DSM-5) also allows for individuals impaired by manic episodes without depression to still be diagnosed with BDI (American Psychiatric Association, 2013). In comparison, a diagnosis of BD type II (BDII) is based on the occurrence of at least one depressive and one hypomanic episode during the lifetime, but no manic episodes (Fig. 1). A diagnosis of BD not otherwise specified or BD unspecified may be given when a patient has bipolar symptoms that do not fit within these major subtype categories. The DSM-5 also includes specifiers which define the clinical features of episodes and the course of the disorder, namely, anxious distress, mixed features, rapid cycling, melancholic features, atypical features, psychotic features (mood-congruent and mood-incongruent), catatonia, peripartum onset, and seasonal pattern (American Psychiatric Association, 2013). In addition, the DSM-5 includes schizoaffective BD as a distinct diagnosis wherein individuals suffer from psychotic symptoms as well as episodes of mania or depression (American Psychiatric Association, 2013).

## Epidemiology

BD is projected to affect about 50 million people worldwide (GBD 2016 Disease and Injury Incidence and Prevalence Collaborators, 2017). The BD subtypes each have an estimated lifetime prevalence of approximately 1% (Merikangas et al., 2007, 2011) although large ranges in lifetime prevalence have been reported (BDI: 0.1–1.7%, BDII: 0.1–3.0%) (Angst, 1998; Merikangas et al., 2007, 2011). Most studies report no gender differences in the prevalence of BD; however, women may be at increased risk for BDII, rapid cycling, and mixed episodes (Diflorio & Jones, 2010; Nivoli et al., 2011). The mean age of onset of BD is at approximately 20 years. An earlier age of onset is associated with poorer prognosis, increased comorbidity, onset beginning with depression, and more severe depressive episodes, as well as longer treatment delays (Joslyn, Hawes, Hunt, & Mitchell, 2016). Additionally, initial depressive episodes may lead to a misdiagnosis of major depressive disorder until the onset of manic or hypomanic episodes necessary to confirm BD (Zimmerman, Ruggero, Chelminski, & Young, 2008).

BD is often comorbid with other psychiatric (Eser, Kacar, Kilciksiz, Yalçinay-Inan, & Ongur, 2018; Frías, Baltasar, & Birmaher, 2016; Salloum & Brown, 2017) and non-psychiatric disorders (Bortolato, Berk, Maes, McIntyre, & Carvalho, 2016; Correll et al., 2017; Vancampfort et al., 2016). It is estimated that >90% of BD patients have at least one lifetime comorbid disorder, and >70% present with three or more comorbid disorders during their lifetime (Merikangas et al., 2007). Such ubiquitous comorbidity within BD suggests the disturbance of multiple systems and pathways, and the presence of comorbidities is associated with increased premature mortality in BD when compared to the general population (Kessing, Vradi, McIntyre, & Andersen, 2015; Roshanaei-Moghaddam & Katon, 2009).

## Classical genetic epidemiology

### Family studies

Genetic factors for BD were first investigated using twin, family, and adoption studies. These studies established that family history of BD is an important clinical predictor of The onset of mood disorders in a patient and that the risk of mood disorder decreases as the genetic distance from the proband increases (Craddock & Sklar, 2013; Merikangas & Yu, 2002). A large family-based Swedish study showed the risk of BD was as much as 7.9, 3.3, and 1.6 times higher for first-, second-, and third-degree relatives of BD probands, respectively, than those without a proband in their family (Song et al., 2015). In the largest family study to date in the Swedish cohort with over 2 million individuals, the transmission of BD was found to be statistically homogenous across family type (intact family, families without fathers, and adoptive families) (Kendler, Ohlsson, Sundquist, & Sundquist, 2020). This family-based study also estimated the heritability of BD, which is a measure of the proportion of variation in a given trait attributed to genetic variation, to be 44% (95% CI 36–48%). Estimates of heritability from twin studies, which compare the concordance of disease between monozygotic and dizygotic twins, were between 60% and 90% (Craddock & Sklar, 2013; Merikangas & Yu, 2002; Smoller & Finn, 2003). Furthermore, by comparing estimates from twin studies, the heritability of BD is among the highest of all other psychiatric and behavioral disorders (Bienvenu, Davydow, & Kendler, 2011).

It has also been well-established that familial risk of BD correlates with increased familial risk of other psychiatric disorders such as schizophrenia, depression, anxiety, drug abuse, attention-deficit hyperactivity disorder (ADHD), personality disorders, and autism spectrum disorder (ASD) (Craddock & Sklar, 2013; Kendler et al., 2020; Song et al., 2015). Schizophrenia, ASD, and depression have the strongest genetic correlations with BD as identified through family studies.

### Cohort and population studies

Sample sizes of studies have rapidly increased as genetic studies of BD have moved from family-based designs to cohort and population-based designs. With these, computationally-efficient methods, such as linkage disequilibrium score regression (LDSC), have been developed to estimate both heritability and genetic correlation captured by the single nucleotide polymorphisms (SNPs) which are common locations in the genome where variation occurs between individuals and are measured on a genotyping array (Bulik-Sullivan et al., 2015a).

**Fig. 2.**
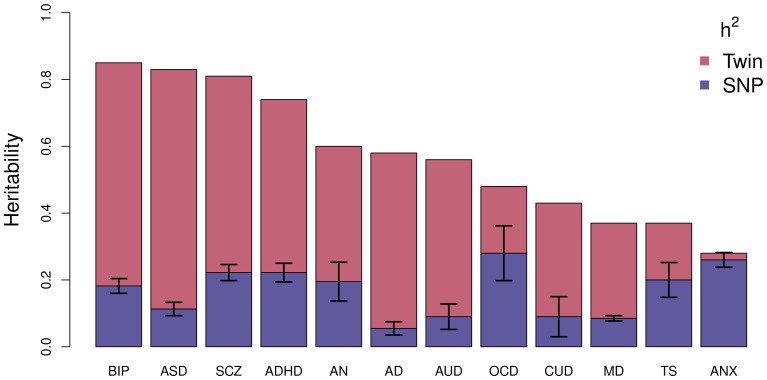
Estimated heritability of psychiatric, behavioral, and neurological disorders. Mean twin-based and SNP-based (on liability scale) heritability for different psychiatric (BIP, bipolar disorder; SCZ, schizophrenia; ADHD, attention-deficit/hyperactivity disorder; MD, major depression; ANX, generalized anxiety disorder), behavioral (AN, anorexia nervosa; AUD, alcohol use disorder; CUD, cannabis use disorder), or neurological (ASD, autism spectrum disorder; AD, Alzheimer's disorder; OCD, obsessive-compulsive disorder; TS, Tourette's syndrome) disorders. Error bars are shown for SNP-based estimates from LDSC.

Using this technique, the largest study of BD estimated an SNP-based heritability (*h*^2^_SNP_), which is a measure of the proportion of variation in a given trait attributed to the genetic variation captured by a genotyping array, of 18.6% (Mullins et al., 2020). Figure 2 compares the latest estimates of the twin-based heritability and *h*^2^_SNP_ for BD to a range of psychiatric, behavioral, and neurological disorders (Bienvenu et al., 2011; Browne, Gair, Scharf, & Grice, 2014; Cross-Disorder Group of the Psychiatric Genomics Consortium, 2019; Demontis et al., 2019a; Faraone & Larsson, 2019; Gatz et al., 2006; Jansen et al., 2019; Purves et al., 2020; Walters et al., 2018; Zilhão et al., 2017). BD has the greatest twin-based heritability estimate and, similar to other traits, also has a substantial proportion not captured by common variations.

New methodologies have also allowed the genetic correlation between different traits to be estimated using the summary statistics from overlapping sets of common SNPs in different samples. A recent cross-disorder analysis of eight different psychiatric and neurological disorders found that BD is most strongly genetically correlated with schizophrenia (*r*_g_ = 0.70) but also shares substantial genetic overlap with major depression (*r*_g_ = 0.36), obsessive-compulsive disorder (OCD; *r*_g_ = 0.31), anorexia nervosa (*r*_g_ = 0.21), ADHD (*r*_g_ = 0.14), and ASD (*r*_g_ = 0.14) (Cross-Disorder Group of the Psychiatric Genomics Consortium, 2019). Furthermore, the high comorbidity of substance use in BD has been linked to substantial genetic correlation with substance use: cigarette use (*r*_g_ = 0.1), cannabis use (*r*_g_ = 0.27), and alcohol use disorder (*r*_g_ = 0.30) (Jang et al., 2020; Kranzler et al., 2019).

## Molecular genetic epidemiology

### Common variants

Genome-wide association studies (GWASs) have been the most successful strategy for identifying specific genetic variants associated with BD. Unlike linkage studies, a GWAS can be performed on a set of unrelated cases and controls by testing for the association of genetic variants across the genome with a trait. The first GWAS of BD in 2007 included 2000 cases and 3000 controls and only identified one independent association signal at *p* < 5 × 10^–7^ (Wellcome Trust Case Control Consortium, 2007). This association was below the now established *p* < 5 × 10^–8^ threshold for genome-wide significance and further, this association was not supported in an expanded reference group analysis nor in an independent replication. Since then, dozens of additional GWASs of case–control cohorts, consisting of samples from mostly European ancestries, have been published (Table 1). Until the sample size of the GWASs increased to over 10 K, these GWASs identified very few genome-wide significant loci. The most recent GWAS was based on a meta-analysis of 52 case–control cohorts and five large population-based cohorts and included over 40 K cases and 350 K controls identified 64 independent loci across the genome associated with BD (Mullins et al., 2020). Thirty-three of these loci were novel for BD, including the region containing the major histocompatibility complex which is also strongly associated with schizophrenia (Mullins et al., 2020; Schizophrenia Working Group of the Psychiatric Genomics Consortium, 2014). Furthermore, additional novel loci identified in this GWAS are also reported as risk loci for schizophrenia, depression, childhood-onset psychiatric disorders, and problematic alcohol use, highlighting shared underlying genetic architecture between BD and these other psychiatric disorders (Mullins et al., 2020). Still though, the SNPs in this large study only explain 15–18% of the variance in the trait (Mullins et al., 2020).

**Table 1.** Summary of bipolar disorder GWAS

Reference	Discovery sample (*n* case + *n* control)	Replication sample (*n* case + *n* control)	Number of genome-wide significant loci	Ethnicity
(Wellcome Trust Case Control Consortium, 2007)	2000 + 3000	–	0	European
(Baum et al., 2008; Ferreira et al., 2008)	461 + 562	772 + 876	1	European
(Ferreira et al., 2008)	4387 + 6209	–	1	European
(Sklar et al., 2008)	1461 + 2008	365 + 351	0	European
(Hattori et al., 2009)	107 + 107	395 + 409	0	Japanese
(Scott et al., 2009)	2076 + 1676	1868 + 12 831	0	European
(Smith et al., 2009)	1001 + 1033	–	0	European
	345 + 670	–	0	African-American
(Djurovic et al., 2010)	194 + 336	435 + 10 258	0	European
(Cichon et al., 2011)	682 + 1300	1729 + 2313 6030 + 31 749	1	European
(Kerner, Lambert, &Muthén, 2011)	1000 + 1034	–	2	European
(Psychiatric GWAS Consortium Bipolar Disorder Working Group, 2011) – PGC1 BD	7481 + 9250	4496 + 42 422	2	European
(Smith et al., 2011)	1190 + 401	2191 + 1434	0	European
(Yosifova et al., 2011)	188 + 376	122 + 328	0	European
(Bergen et al., 2012)	836 + 2093	–	0	European
(Chen et al., 2013)	6658 + 8187	1115 + 2728	5	European + Taiwanese
(Lee, Woo, Greenwood, Kripke, & Kelsoe, 2013)	2191 + 1434	–	0	European
(Kuo et al., 2014)	200 + 200	351 + 341	0	Taiwanese
(Mühleisen et al., 2014)	9747 + 14 278	–	5	European
(Xu et al., 2014)	950 + 950	–	0	European
(Hou et al., 2016)	7647 + 27 303	2137 + 3168	6	European
(Kao et al., 2016)	189 + 1773	283 + 500	0	Taiwanese
(Charney et al., 2017)	13 902 + 19 279	–	8	European
(Ikeda et al., 2018)	2964 + 61 887	–	1	Japanese
	10 445 + 71 137	–	5	Japanese + European
(Fiorica & Wheeler, 2019)	359 + 686	–	0	African-American
(Stahl et al., 2019) – PGC2 BD	20 352 + 31 358	9412 + 137 760	30	European
(Mullins et al., 2020) – PGC3 BD	41 917 + 371 549	–	64	European

**Fig. 3.**
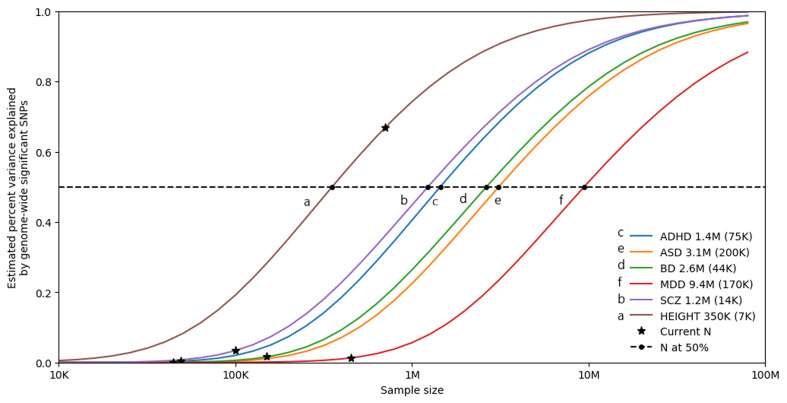
Statistical power calculations for current and future GWAS. The variance explained by genome-wide significant variants (*y*-axis) is calculated for increasing GWAS sample sizes (*x*-axis) using the univariate causal mixture model (Holland et al., 2020). The legend describes the estimated GWAS sample sizes (SE) needed to capture 50% of the genetic variance (horizontal dashed line) associated with each trait. Stars indicate the sample sizes of currently available GWAS, and circles indicate the estimated sample sizes needed to capture 50% of the genetic variance for each trait. Traits include attention-deficit/hyperactivity disorder (ADHD) (Demontis et al., 2019b), autism spectrum disorder (ASD) (Grove et al., 2019), bipolar disorder (BD) (Mullins et al., 2020), depression (MDD) (Howard et al., 2019), and schizophrenia (SCZ) (Pardiñas et al., 2018). Height is included as a somatic control (no genetic correlation exists between height and bipolar disorder) (Yengo et al., 2018). s.e., standard error.

Furthermore, as shown in Fig. 3, univariate causal mixture modeling suggests that we can expect to see substantial increases in identified genome-wide significant loci and consequently in the proportion of *h*^2^_SNP_ explained by these variants as GWAS sample sizes increase (Holland et al., 2020). This is particularly relevant for BD where GWAS studies have now reached the ‘inflection’ point where the significant associations begin to accumulate with smaller increases in sample size (Mullins et al., 2020). As such, international collaborations in large-scale GWAS remain imperative for the continued identification of common variants underlying BD etiology, and the plan of the PGC Bipolar Working Group to further increase GWAS sample sizes is encouraging (Sullivan et al., 2018).

### Genetic overlap

In addition to genetic correlation (Bulik-Sullivan et al., 2015b) (described above), the most common approach for assessing genetic overlap at the genome-wide level is polygenic risk score (PRS) analysis (International Schizophrenia Consortium et al., 2009). The PRS for a given trait is typically a weighted sum of genetic variants where the variants used and their weights are defined by effects measured by previous GWASs of the trait. The genetic liability for BD has been used to predict a number of other psychiatric disorders as well as creativity, educational attainment (Mistry, Harrison, Smith, Escott-Price, & Zammit, 2018), addiction (Reginsson et al., 2018), as well as psychopathology (Mistry, Escott-Price, Florio, Smith, & Zammit, 2019a), cognitive functioning (Mistry, Escott-Price, Florio, Smith, & Zammit, 2019b), progression of unipolar to bipolar depression, and depression onset (Musliner et al., 2019, 2020).

PRSs for BD and other traits have also been used to explain common comorbidities within BD. Suicide attempts by people with BD have been associated with higher genetic liability for depression (Mullins et al., 2019) as well as an interaction between trauma and bipolar genetic liability (Wilcox et al., 2017). Previous childhood ADHD diagnosis in those with BD was associated with higher genetic liability for ADHD (Grigoroiu-Serbanescu et al., 2020; Wilcox et al., 2017).

In addition to PRS analysis, cross-disorder GWAS meta-analyses have also been performed for BD and ADHD (Bipolar Disorder and Schizophrenia Working Group of the Psychiatric Genomics Consortium, 2018; van Hulzen et al., 2017), SCZ (Bipolar Disorder and Schizophrenia Working Group of the Psychiatric Genomics Consortium, 2018), and MDD (Coleman, Gaspar, Bryois, & Breen, 2020), identifying two, 114, and 73 genome-wide significant loci associated with these phenotype pairs, respectively. Moreover, numerous genes mapped to BD risk loci are also linked to schizophrenia, ASD, and OCD (O'Connell, McGregor, Lochner, Emsley, & Warnich, 2018) further highlighting common genetic architecture across psychiatric disorders.

The conditional/conjunctional false discovery rate statistical tool has also been used to identify BD risk loci as well as shared risk loci between BD and a number of phenotypes. This method leverages the power of two GWAS to boost discovery by re-adjusting the GWAS test statistics in a primary phenotype and allows for the discovery of loci significantly associated with two phenotypes simultaneously (Andreassen, Thompson, & Dale, 2014; Smeland et al., 2020b). Utilizing this method, shared genetic loci have been identified between BD and ADHD (*n* = 5) (O'Connell et al., 2019), schizophrenia (*n* = 14) (Andreassen et al., 2013), Alzheimer's disease (*n* = 2) (Drange et al., 2019), intelligence (*n* = 12) (Smeland et al., 2020a), body mass index (*n* = 17) (Bahrami et al., 2020), and lifespan (*n* = 8) (Muntané et al., 2021). This method is agnostic to the effect directions of genetic variants and so shared loci were identified between BD and Alzheimer's disease, intelligence, body mass index, and lifespan despite observed null and non-significant genetic correlations with these phenotypes.

Most recently (Mullins et al., 2020), the genetic relationship between BD and 10 clinically and epidemiologically associated traits (daytime sleepiness, morningness, sleep duration, insomnia, mood instability, educational attainment, problematic alcohol use, drinks per week, smoking initiation, and cigarettes per day) were assessed using the MiXeR tool (Frei et al., 2019), to identify trait-specific and shared genetic components, and Mendelian randomization (Zhu et al., 2018), to establish ‘causal’ relationships. Extensive genetic overlap was identified between all traits and BD, most notably that >90% of the genetic variants estimated to influence BD were also estimated to influence educational attainment. Moreover, bidirectional relationships were identified between BD and sleep duration, mood instability, educational attainment, and problematic alcohol use, while BD was identified as ‘causal’ for morningness and drinks per week and smoking initiation was ‘causal’ for BD (Mullins et al., 2020).

### Rare variants

In addition to genetic interactions, the difference in heritability could also be explained by rare variants in the genome which are often unmeasured and thus not included in GWASs. While the cost of whole-exome sequencing (WES) and whole-genome sequencing (WGS) has decreased, these technologies are still substantially more expensive than common genotyping arrays. As a result, WGS/WES studies of BD have been limited to small studies consisting mostly of large pedigrees to potentially enrich the sample with causal rare variants and increase power (Forstner et al., 2020; Goes et al., 2016, 2019; Maaser et al., 2018; Sul et al., 2020; Toma et al., 2018). While these studies have found evidence of higher rare deleterious burden in cases (Sul et al., 2020), higher disruptive variant burden in early-onset cases (Toma et al., 2018), evidence of rare variant segregation in pedigrees (Forstner et al., 2020; Goes et al., 2016; Maaser et al., 2018), and evidence of *de novo* variation (Goes et al., 2019), much larger sample sizes will be required to definitively identify rare variants conferring risk for BD.

### Copy number variants

Copy number variants (CNVs) refer to regions of the genome where a duplication (three or more copies are present) or deletion (only one copy remains) has occurred such that more or less than the expected two copies in the diploid human genome are present. Carriers of certain CNVs are known to be at considerably elevated risk for developing neurodevelopmental (e.g. ASDs) and mental disorders (e.g. schizophrenia) (Kirov, Rees, & Walters, 2015) as well as somatic conditions (e.g. diabetes and hypertension) (Crawford et al., 2019). The frequency of CNVs in BD is less than that observed for neurodevelopmental disorders or schizophrenia (Kirov, 2015), and correspondingly their role in the disorder appears less with only one CNV robustly associated with BD to date. A 650 kb duplication at 16p11.2 was first described as a *de novo* CNV for BD (Kirov, 2015; Malhotra et al., 2011) and this association was replicated in a larger genome-wide analysis (Green et al., 2016). This CNV is also implicated in schizophrenia, autism, and intellectual disability (Kirov, 2015). Two additional CNVs, at 1q21.1 and 3q29, are also implicated in BD; however, these associations fail to pass the genome-wide significance threshold (Green et al., 2016). Interestingly, these two CNVs are also associated with schizophrenia (Kirov, 2015). One further study identified enrichment of genic CNVs in schizoaffective BD, but not between BD cases and controls or other BD subtypes (Charney et al., 2019).

These findings highlight that the genetic overlap between BD and schizophrenia extends beyond common variation, but suggests a difference in underlying mechanisms. One possible explanation for the smaller role of CNVs in BD is that patients with BD exhibit less cognitive deficits than patients with schizophrenia who can exhibit substantial cognitive deficits, since the same CNVs which are implicated in schizophrenia are also known to cause cognitive problems (Kirov, 2015).

### Genetic interactions

Other than increasing the sample size of GWAS, the difference between observed twin-based and *h*^2^_SNP_ (Fig. 2) may also be explained by unaccounted for moderated genetic effects such as interactions between genes and the environment (G×E) or gene–gene interactions (epistatic effects). The role of G×E in BD remains an under-researched area, however, but some interactions have been identified (Aas et al., 2014, 2020; Hosang, Fisher, Cohen-Woods, McGuffin, & Farmer, 2017; Oliveira et al., 2016; Winham et al., 2014). Although these studies highlight the potential role of G×E in the etiology of BD, the lack of replication studies and small sample sizes suggest that they should be interpreted with caution. As with G×E, studies of epistasis in BD are in their infancy and lack replication (Judy et al., 2013). As the ability of GWAS to identify risk variants with small effects increases, further study of how implicated genes interact with environmental or other genetic factors to modulate the risk of BD are required.

## Clinical implications

### Pharmacogenomics

Lithium, anti-epileptic drug mood stabilizers (such as valproate/divalproex, lamotrigine, and carbamazepine), antipsychotics, and antidepressants are commonly prescribed treatments for BD. However, response to these medications can widely vary between individuals, and some patients may cycle through different medications before they find an effective treatment with minimal side effects. Pharmacogenomic studies aim to use genetics to predict treatment response. A particular challenge to pharmacogenomics in BD has been the measurement of treatment response which can be limited by the length of follow-up, adherence to medication, and confounding due to the multi-drug treatment strategy common to the illness. Consequently, a systematic rating system with a high inter-rater reliability, the Alda score, was developed to quantify the clinical improvement of BD during treatment while also accounting for potential confounders of treatment response (Nunes, Trappenberg, & Alda, 2020). However, obtaining large samples with reliable measures has limited the statistical power to discover clinically-informative genetic variants associated with treatment response. Furthermore, heterogeneity between study designs and the samples included have yielded limited replication of any findings. While not yet replicable, promising pharmacogenomic findings for BD were summarized in a recent review (Gordovez & McMahon, 2020). Most of the previous pharmacogenomic studies have been focused on either lithium treatment response or HLA haplotypes predicting serious adverse reactions related to carbamazepine, phenytoin, and lamotrigine. A recent study tested for genetic association with treatment response to anti-epileptic drug mood stabilizers, an alternative to lithium, and identified two SNP-level associations in *THSD7A* and *SLC35F3* as well as two gene-level associations with *ABCC1* and *DISP1* (Ho et al., 2020).

With the exception of genetic predictors of adverse reactions to medication, no large genetic effects on treatment response have been identified. However, current pharmacogenomic testing has already been shown to be useful by providing clinicians support in reaching effective and well-tolerated treatments of BD (Ielmini et al., 2018). Additionally, as the sample size of pharmacogenomic studies increases, PRSs derived from these studies could further enable a precision medicine approach to BD treatment. In addition to pharmacogenomic PRSs, PRSs derived from large case–control studies could also improve the genetic prediction of treatment response. For example, increased genetic liability for depression and schizophrenia was associated with worse response to lithium (Amare et al., 2020; International Consortium on Lithium Genetics (ConLi+Gen) et al., 2018). These PRSs could be explaining some of the clinical heterogeneity in the sample as discussed below and thus improve the identification of certain BD clinical profiles that respond best to lithium.

Finally, there is potential application of repurposing drugs and focusing on different drug targets based on recent genetic findings. For example, calcium channel blockers (CBBs), which have been widely used to treat hypertension and other cardiovascular conditions, were also once considered as a treatment in psychiatry (Harrison, Tunbridge, Dolphin, & Hall, 2020). However, because CACNA1C has now been implicated as one of the strongest associations with BD (Gordovez & McMahon, 2020), there is renewed interest in CBBs as a treatment for the disorder (Cipriani et al., 2016).

### Risk prediction

In addition to therapeutic intervention, PRSs may also provide clinical utility to inform disease screening (Torkamani, Wineinger, & Topol, 2018). While the PRS derived from the latest GWAS of BD only explains about 4.75% of the phenotypic variance, the latest PRS could still be useful for risk stratification (Mullins et al., 2020). Compared to individuals with average genetic risk for BD, individuals in the top decile risk had an odds ratio of 3.62 (95% CI 1.7–7.9) of being a case. An important caveat to note about PRSs, however, is that prediction performance is worse when applied to ancestries not included in the training GWAS (Martin et al., 2019). For instance, the current BD PRS, estimated using individuals with European ancestries, explains only around 2% and 1% of the phenotypic variance in individuals with East Asian or admixed African American ancestry, respectively (Mullins et al., 2020). Encouragingly though, the trans-ethnic prediction accuracy of the PRS has improved as the sample size has increased. Furthermore, the PRS prediction accuracy will also improve as new non-European ancestries are included in future training GWASs.

### Clinical heterogeneity

PRSs can also help dissect the high clinical heterogeneity (i.e. bipolar type, psychosis, rapid cycling) present in the disorder (Coombes et al., n.d.). For example, higher genetic liability for schizophrenia is associated with bipolar type I (Charney et al., 2017). This finding could be driven by the increased prevalence of psychosis among those with BDI as multiple studies have shown that higher genetic risk of schizophrenia is associated with psychosis in BD, particularly during mania (Allardyce et al., 2018; Bipolar Disorder and Schizophrenia Working Group of the Psychiatric Genomics Consortium, 2018; Charney et al., 2019; Coombes et al., 2020; Markota et al., 2018). Other studies of bipolar subtypes have shown positive associations between BDII and insomnia PRS, rapid cycling and ADHD PRS, as well as early age-of-onset of BD and PRSs for risk-taking and anhedonia (Coombes et al., n.d.; Lewis et al., 2019). While no individual PRS is able to explain a large amount of variation among bipolar subtypes, these findings give insight into the genetic contributions to clinical heterogeneity and could help classify the disorder more accurately as well as identify the risk of suicide, psychosis, and other adverse outcomes in patients with BD.

### Future directions

Significant advances in our understanding of the genetic architecture of BD have been made, from initial linkage and family studies to current large consortia-driven genome-wide studies. Moreover, integration of these genetic discoveries with other -omic and imaging data will be key to comprehending the role of genetic variation in the etiology of BD. However, distinct shortcomings and limitations to genetic discovery highlight key areas to be prioritized in future studies.

### Diverse phenotype ascertainment

Identification of novel loci for BD, and other polygenic complex phenotypes, requires increasing sample sizes (Fig. 3), which remains a challenging and costly task (Lu, Campeau, & Lee, 2014). The majority of samples included in the PGC-BD were clinically ascertained, with the inclusion of external biobank samples only in the most recent discovery GWAS (Mullins et al., 2020). Numerous efforts have been made to combine electronic health record and registry data with genetic data to facilitate large population-based studies, such as the Electronic Medical Records and Genomics network (https://emerge-network.org/), the UK Biobank (https://www.ukbiobank.ac.uk/), All of Us (https://allofus.nih.gov/), the Million Veterans Program (https://www.research.va.gov/mvp/), and iPsych (https://ipsych.dk/en/). Furthermore, GWAS summary statistics of self-reported phenotypes for thousands to millions of individuals may be obtained through collaboration with the personal genetics company 23andMe, Inc. (https://research.23andme.com/research-innovation-collaborations/). The data generated by such population studies and 23andMe provide a means by which to drastically increase sample size without the costs associated with clinical ascertainment. This approach was shown to be successful for depression, where PGC cohorts were meta-analyzed with data from the UK Biobank and summary statistics from 23andMe, increasing the number of identified associated risk loci from 44 (Wray et al., 2018) to 102 (Howard et al., 2019). However, a limitation to this use of ‘minimal phenotyping’ data is that the loci identified, especially when based on self-report data, were non-specific for depression highlighting potential differences in genetic architecture when compared to clinically ascertained depression (Cai et al., 2020). In line with this, the *h*^2^_SNP_ estimates of the biobank samples included in the latest PGC BD GWAS are less than that observed for clinically ascertained samples which may reflect more heterogeneous clinical presentations or less severe illness (Mullins et al., 2020).

Data generated from ‘minimal phenotyping’ are likely to include other psychopathological features which may underlie self-reported BD such as personality disorders or mild temperamental traits, thereby increasing heterogeneity in the sample and leading to the possibility of non-specific or false-positive results. However, true self-reported BD may reflect the non-hospitalized, non-psychotic part of the BD spectrum, more typical of BDII, which is under-represented in the current PGC BD sample. Moreover, expanding genetic studies to include the full spectrum of BD in population-based non-clinical samples increases the potential for novel discoveries with important implications for clinical management and further research, and is therefore of high interest to both clinicians and the pharmaceutical industry.

Thus, while adopting the ‘minimal phenotyping’ approach for BD will allow GWAS to reach sample sizes not currently feasible by clinical ascertainment and will likely identify numerous novel risk loci, similar post-hoc analyses as that performed for depression (Cai et al., 2020), will be required to determine the specificity of identified loci to BD.

### Increased deep phenotyping

The high levels of heterogeneity amongst patients with BD, including disorder type, features of episodes, and the course of the disorder, contribute to the difficulty in identifying underlying genetic risk factors. BDI (*h*^2^_SNP_ = 25%) is shown to be more heritable than BDII (*h*^2^_SNP_ = 11%), and the genetic correlation (*r*_g_ = 0.89) between these types suggests that they are closely related, yet distinct, phenotypes (Stahl et al., 2019). In support of this, the most recent PGC GWAS for BD identified novel and distinct loci specifically associated with BDI or BDII, which were not identified when all bipolar cases were analyzed together (Mullins et al., 2020). Genetic studies of the features and course of BD have predominantly employed a PRS approach, as outlined above, and GWAS data for these subtypes is lacking due to small sample sizes [data from the PGC indicate that none of these subtypes include more than 10 K samples (Bipolar Disorder and Schizophrenia Working Group of the Psychiatric Genomics Consortium, 2018)]. Thus, larger deeply phenotyped samples are required in order to conduct a thorough investigation of the genetic architecture of these subtypes within BD. Doing so would aid subtype-specific discoveries, and may inform on nosology, diagnostic practices, and drug development for BD.

In addition, the potential inclusion of ‘minimal phenotyping’ data, as described above, further emphasizes the need for increased deep phenotyping. Results generated from deep phenotyped samples will serve as standards against which to compare the specificity of results generated from the inclusion of ‘minimal phenotyping’.

### Increased ancestral-diversity

The majority of individuals included in GWASs for any trait have overwhelmingly been of European descent and the lack of diversity is even more pronounced in genetic studies of psychiatric disorders (Martin et al., 2019; Peterson et al., 2019; Sirugo, Williams, & Tishkoff, 2019). In BD, the largest GWAS includes only individuals from European ancestries (Mullins et al., 2020). This ‘missing diversity’ can greatly hinder our understanding of the etiology of BD. For example, the inclusion of non-European ancestries could substantially improve fine-mapping of disease-associated loci (Peterson et al., 2019). Furthermore, the current Eurocentric approach has the potential to exacerbate health disparities already seen in BD (Akinhanmi et al., 2018) by limiting the therapeutic advances gained by pharmacogenomics and improved genetic risk predictions to those of European descent (Duncan et al., 2019; Martin et al., 2019; Sirugo et al., 2019). Future inclusion of diverse samples will come with new ethical, technological, and methodological challenges (Peterson et al., 2019). Some of these considerations include choosing ancestry-specific genotyping platforms to improve genomic coverage, increasing sample sizes of diverse reference panels to improve imputation accuracy, and improving statistical methods to control for population stratification and estimate ancestry-specific PRSs. Thus, the PGC Bipolar Working Group has committed to expanding the future GWAS to include non-European ancestries.

### Larger sequencing efforts

As mentioned above, sequencing efforts in BD are currently in their infancy (Forstner et al., 2020; Goes et al., 2016; Maaser et al., 2018; Sul et al., 2020; Toma et al., 2018). Although studies provide evidence that rare variants might contribute to the etiology of BD, weak statistical power due to small sample sizes remains an issue. The Bipolar Sequencing Consortium (BSC) was established to facilitate combining existing exome and WGS studies of BD (http://metamoodics.org/bsc/consortium/), and includes approximately 4500 BD cases and 9000 controls, as well as 1200 affected relatives from 250 families. Moreover, a collaboration between the Dalio Initiative in BD (https://www.daliophilanthropies.org/initiatives/mental-health-and-wellness/), the Stanley Centre (https://www.broadinstitute.org/stanley), and iPSYCH (https://ipsych.dk/en/) aims to generate WES data from approximately 7000 BD cases and 10 000 matched controls. However, it is estimated that as many as 25 000 cases might be necessary in order to identify significant rare variant associations with BD (Zuk et al., 2014), confirmed by recent analyses in schizophrenia (Singh et al., 2020), and so continued expansion of these, or similar, efforts will be crucial to determine the role of rare variation in BD.

## Conclusion

Our knowledge of the genetic etiology of BD has rapidly accelerated in recent years with advances in technology and methodology as well as the adoption of international consortiums and large population-based biobanks. It is now clear that BD is highly heritable but also highly heterogeneous and polygenic with substantial genetic overlap with other psychiatric disorders. Encouragingly, genetic studies of BD have reached an ‘inflection point’ (Fig. 3). Thus, the number of associated loci is expected to substantially increase in larger future studies and with it, improved genetic prediction of the disorder. Incorporation of ancestrally-diverse samples in these studies will enable improved identification of causal variants for the disorder and also allow for equitable future clinical applications of both genetic risk prediction and therapeutic interventions.
